# Molecular Profiling of Acute Myeloid Leukemia in Pakistan: Comprehensive Variant Landscape Revealed by Targeted NGS

**DOI:** 10.3390/ijms27093927

**Published:** 2026-04-28

**Authors:** Rafia Mahmood, Saleem Ahmed Khan, Sadia Ali, Fatima Sharif, Umar Khurshid, Dilshad Ahmed, Eshal Shahzad, Hamid Saeed Malik, Naghmi Asif, Sidrah Jahangir

**Affiliations:** 1 Armed Forces Institute of Pathology, Rawalpindi 46000, Pakistan; dr.sai@hotmail.com (S.A.); fatimasharif425@gmail.com (F.S.); umar_kim@yahoo.com (U.K.); hamidsaeed_malik@yahoo.com (H.S.M.); sidrahjahangir1@gmail.com (S.J.); 2 National University of Medical Sciences, Abid Majeed Road, Rawalpindi 46000, Pakistan; saleem.mc235@gmail.com (S.A.K.); dakhan@cpsp.edu.pk (D.A.); 3 Islamabad Medical and Dental College, Islamabad 44000, Pakistan; shahzadeshal2005@gmail.com (E.S.); naheed.naghmi@imdcollege.edu.pk (N.A.)

**Keywords:** acute myeloid leukemia (AML), next-generation sequencing (NGS), somatic mutations, genomic profiling, co-mutation analysis, variant allele frequency (VAF), precision oncology

## Abstract

Acute myeloid leukemia (AML) is a heterogeneous malignancy, with clonal complexity and somatic mutations critically influencing prognosis and treatment. While global genomic profiling efforts have revolutionized AML classification and risk stratification, the molecular landscape in Pakistani patients remains underexplored. Our aim is to perform targeted next-generation sequencing (NGS) for somatic mutational profiling of newly diagnosed AML patients in Pakistan. This prospective study was conducted at the Armed Forces Institute of Pathology, Pakistan, from January 2021 to January 2026. Among 104 patients, 204 somatic variants were identified (mean: 1.96 variants/patient), predominantly single-nucleotide variants (49.5%). Missense mutations (38.2%) were most common, with enriched transitions (Ti/Tv: 1.27:1). Frequently mutated genes included *TP53* (22.1%), *KIT* (9.8%), *CEBPA* (8.8%), and *NRAS* (5.9%). Cell-signaling genes (30.4%) and tumor suppressor genes (27.0%) were the most affected functional groups. Co-mutation analysis showed clustering led by *DNMT3A–IDH1* co-occurrence (ρ ≈ 0.43). DNA-methylation alterations frequently co-occurred with tumor suppressors (OR ≈ 4.6, *p* = 0.007), transcription factors (OR ≈ 3.9, *p* = 0.023), and *NPM1* (ρ = 0.32). This study provides the first comprehensive genomic map of Pakistani AML patients, revealing unique mutational signatures. The findings lay the groundwork for population-specific precision oncology in low- and middle-income countries.

## 1. Introduction

Acute myeloid leukemia (AML) is a hematologic malignancy characterized by the clonal proliferation of myeloid progenitors. It is not one disorder, rather a whole spectrum of disorders, with biologic heterogeneity, phenotypic variability, and diverse clinical courses ranging from aggressive disease to biologically distinct subtypes with more favorable outcomes [1]. Despite therapeutic advances, prognosis remains poor, with five-year overall survival approximating 40% in patients under 60 years and plummeting to 1–5% in those over 70 years [2]. Prognosis is determined by age, comorbidities, AML subtype, but most importantly by the underlying molecular and cytogenetic alterations. In AML, cytogenetic abnormalities are well characterized and essential for disease classification. They play an important role in risk stratification and predicting disease course [3].

Advances over the years and the advent of high-throughput sequencing have uncovered extensive genetic diversity in AML, with over 2000 somatic mutations identified across critical regulatory pathways [4]. These mutations impact core biological processes that govern leukemogenesis, clonal evolution, and therapeutic resistance, thereby reshaping AML classification and risk stratification [5]. Comprehensive genomic profiling at diagnosis, coupled with minimal residual disease monitoring, is now essential for guiding precision oncology and optimizing treatment decisions [6].

AML pathogenesis and disease course are shaped by complex, co-occurring mutations that influence clonal architecture and alter clinical outcomes [7]. Prognosis in AML is shaped by the interaction of multiple genetic alterations; for instance, the favorable impact of *NPM1* mutations is attenuated by co-mutations in *FLT3-ITD*, *DNMT3A*, or *IDH1*, while the benefit of biallelic *CEBPA* mutations may be negated by *IDH2* [8]. These results emphasize the importance of integrated, multi-gene profiling for more accurate prognostication and better-informed treatment choices [9].

Next-generation sequencing (NGS) has revolutionized molecular diagnostics in AML, offering high-throughput, parallel analysis of multiple genes with exceptional sensitivity [8]. While whole-genome and exome sequencing provide broad insight, their routine use is limited by cost and complexity. In contrast, targeted myeloid gene panels deliver clinically actionable data efficiently and cost-effectively, focusing on high-yield genomic regions [10]. These panels have become the standard in AML diagnostics, enabling timely, risk-adapted therapeutic decisions in clinical practice [11].

Despite transformative advances in global AML genomics, the mutational landscape in Pakistan remains largely uncharted. To address this gap, we conducted the first comprehensive somatic mutational profiling of newly diagnosed Pakistani AML patients, generating a population-specific genomic dataset. Our findings will not only illuminate ethnically distinct leukemogenic pathways, but also establish a scalable framework for context-driven cancer genomics in low- and middle-income countries.

## 2. Results

### 2.1. Patient Demographics and Baseline Characteristics

Among 104 newly diagnosed AML patients, 63 were males, while 41 were females, with a male-to-female ratio of 1.5:1. The median age was 32.5 years (range: 5–72). Age distribution was skewed toward younger individuals. Pediatric patients (<15 years) constituted a small but notable subset (8.7%).

### 2.2. Variant Burden

Genomic profiling identified 204 somatic variants (31 unique genes) according to standard guidelines [12]. Mean variant burden was 1.96 with a median of 2 variants per patient (range, 0–6). Variant distribution showed 24 patients (23.1%) with no detectable variant, 23 (22.1%) with 1 variant, 24 (23.1%) with 2 variants, 14 (13.5%) with 3 variants, 7 (6.7%) with 4 variants, 9 (8.7%) with 5 variants, and 3 (2.9%) with 6 variants.

### 2.3. Spectrum of Somatic Variants: Types, Functional Consequences, and Nucleotide Substitution Patterns

The mutational spectrum was dominated by single-nucleotide variants (SNVs; 101, 49.5%) followed by deletions (72, 35.3%), insertions (27, 13.2%), and delins (4, 2.0%). At the consequence level, missense variants were most common (78, 38.2%), alongside a substantial burden of feature-truncating (49, 24.0%) and frameshift variants (46, 22.5%); stop-gained and synonymous variants were infrequent (each 10, 4.9%), while in-frame insertions (6, 2.9%) and splice-donor variants (2, 1.0%) were rare. Nucleotide substitutions were transition-enriched, dominated by C>T transitions (33.7%), with A>C and G>A substitutions each contributing 17.8%, yielding a Ti/Tv ratio of 1.27:1 (transitions, 57.6%).

### 2.4. Chromosomal Distribution of Somatic Alterations

**Figure 1:**
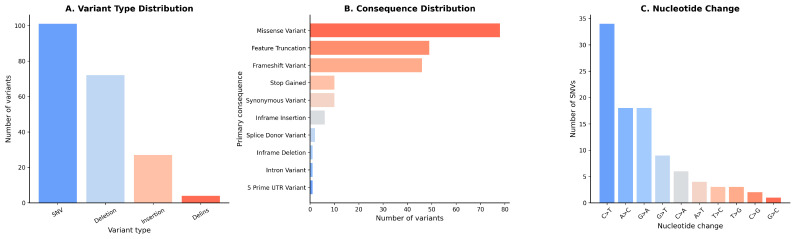
Molecular variant spectrum in 104 AML patients. This multi-panel figure summarizes the distribution and biological impact of somatic variants identified in the study cohort (n = 104 AML patients; total variants = 204). (**A**) Variant type distribution: Bar plot depicting the absolute counts of different variant classes. (**B**) Functional consequence distribution: Horizontal bar plot illustrating the predicted primary consequences of detected variants based on annotation. (**C**) Nucleotide substitution patterns: Bar plot showing the frequency of single-nucleotide substitutions.

**Figure 2:**
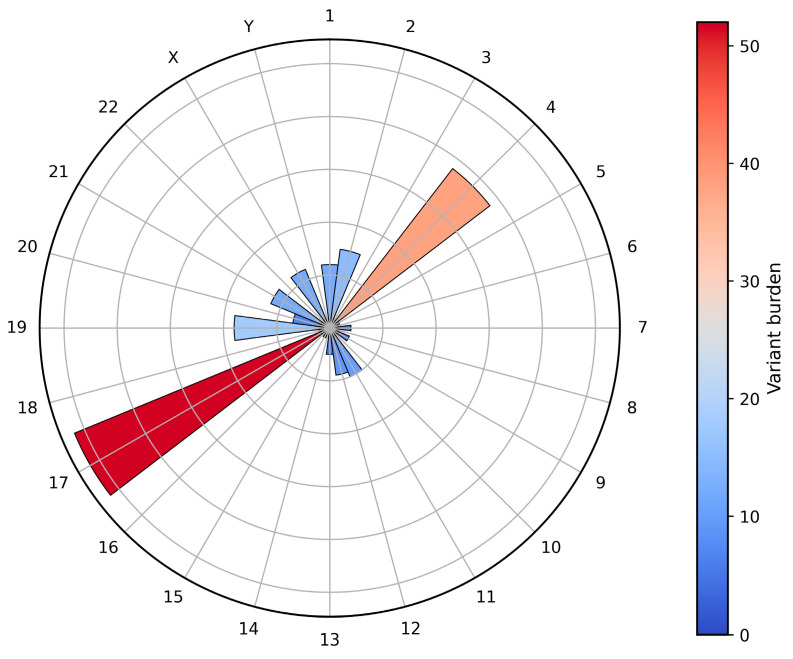
Chromosomal distribution of somatic variants in 104 AML patients. Circular bar plot illustrating the genome-wide distribution of somatic variants across chromosomes (1–22, X, Y) in the study cohort. Each radial bar represents the total variant burden per chromosome, with bar length and color intensity proportional to the number of detected variants.

Chromosomal distribution showed dominant enrichment on chromosomes 17 (22.8%) and 4 (16.7%), together comprising nearly 40% of all events. This pattern was driven by *TP53* alterations on chromosome 17 and recurrent mutations in *KIT*, *PDGFRA*, and *TET2* on chromosome 4, with additional clustering on chromosomes 19 (*CEBPA*), 2 (*DNMT3A*, *IDH1*, *SF3B1*), 21 (*RUNX1*, *U2AF1*), and X (*BCOR*, *PHF6*, *STAG2*, *ZRSR2*). Figure 1 summarizes variant types and consequences, while Figure 2 gives the chromosomal distribution.

### 2.5. Variant Distribution Based on Pathogenicity

Across 104 patients, pathogenic variants accounted for 65 (31.9%) and likely pathogenic variants for 47 (23.0%), together comprising 112/204 (54.9%) of all detected alterations. Within the actionable subset, functional-group stratification showed highest representation of cell-signaling genes (43/112, 38.4%), followed by transcription factors (19/112, 17.0%), DNA methylation (13/112, 11.6%), tumor suppressors (12/112, 10.7%), and epigenetic modifiers (10/112, 8.9%), with smaller contributions from RNA splicing (9/112, 8.0%), cohesin complex (4/112, 3.6%), and *NPM1* (2/112, 1.8%). Among pathogenic and likely pathogenic variants, the most frequently affected genes were *NRAS* (12, 10.7%), *CEBPA* (9, 8.0%), *WT1* (8, 7.1%), *TET2* (7, 6.2%), and *KRAS* (7, 6.2%), followed by *ASXL1*, *U2AF1*, and *IDH1* (each 6, 5.4%), and *RUNX1* and *FLT3* (each 5, 4.5%). Variants of uncertain significance (VUS) represented 64 (31.4%). The VUS category was largely influenced by recurrent deep intronic *TP53* variants (n = 43), particularly NM_000546.6; hg19 chr17:7579644_7579659. These variants lie outside known splice regions and currently lack functional or clinical evidence, suggesting they are most likely benign. Benign (25; 12.3%) and likely benign (3; 1.5%) variants were relatively infrequent and mainly involved *KIT* and *PDGFRA*.

### 2.6. Gene-Level Mutational Profile

**Figure 3:**
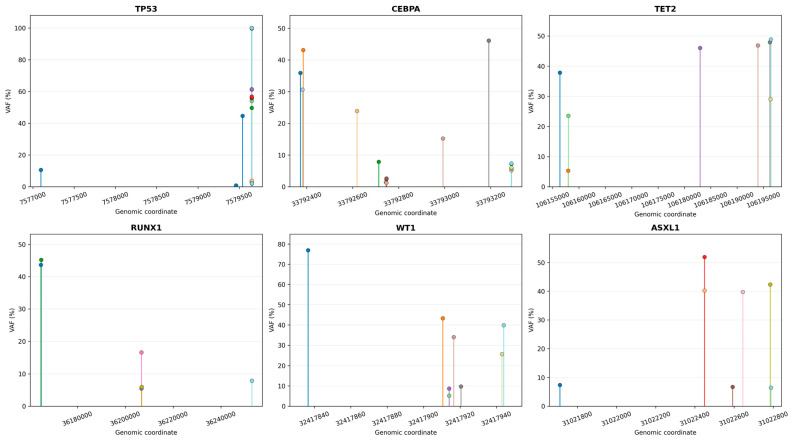
Genomic Distribution of Recurrently Mutated Genes in AML Patients. Lollipop plots illustrating the genomic positions of somatic variants in *TP53*, *CEBPA*, *TET2*, *RUNX1*, *WT1*, and *ASXL1* across the study cohort. Each vertical line represents an individual variant, positioned according to its genomic coordinate (hg19 reference genome), with height corresponding to variant allele frequency (VAF). The plots depict the distribution of mutations within each gene.

Of the 204 variants identified, *TP53* was the most frequently mutated gene (45; 22.1%), followed by *KIT* (20; 9.8%) and *CEBPA* (18; 8.8%). Recurrent mutations were also observed in *NRAS* (12; 5.9%) and *PDGFRA* (10; 4.9%), with additional contributions from *TET2* and *WT1* (each 8; 3.9%). Gene-level analysis revealed marked mutational heterogeneity, with *TP53*, *CEBPA*, *TET2*, and *ASXL1* showing high diversity and low hotspot dominance, in contrast to *NRAS*, *IDH1*, and *JAK2*, which were dominated by recurrent hotspot mutations, while *FLT3* displayed characteristic *ITD/TKD*-driven recurrence. Lollipop plots in Figure 3 illustrate the genomic distribution of unique somatic variants in *TP53*, *CEBPA*, *TET2*, *RUX1*, *WT1*, and *ASXL1*. Each dot represents a unique variant, with vertical stems indicating precise genomic coordinates. Variants are color-coded by pathogenicity.

*FLT3* variants were identified in 5 of 104 patients (4.8%), accounting for 2.5% of all detected variants and 4.5% of pathogenic/likely pathogenic alterations. All *FLT3* variants were classified as likely pathogenic. The mutational spectrum was dominated by exon 14 in-frame insertion variants (60.0%), while exon 20 p.Asp835Tyr (D835Y) mutations accounted for 40.0%. *FLT3* variant allele frequency was heterogeneous (median VAF 0.40; range 0.14–0.81), indicating variable clonal burden across cases.

### 2.7. Functional Genomic Stratification of Somatic Variants Across Biological Pathways and Functional Gene Groups

**Figure 4:**
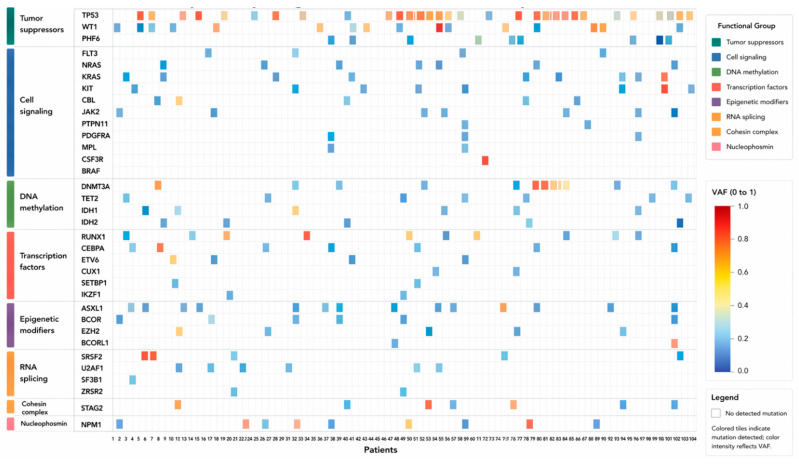
Oncoprint Depicting Somatic Mutational Landscape in 104 AML Patients. Each column represents an individual patient, and each row corresponds to a gene, arranged broadly by frequency of mutation (most frequently mutated genes at the top). Colored tiles indicate the presence of a mutation in a given gene for a specific patient, while blank spaces denote the absence of detectable variants. The intensity of the red color gradient reflects the VAF (range 0–1). Genes are annotated and color-coded according to their functional categories.

**Figure 5:**
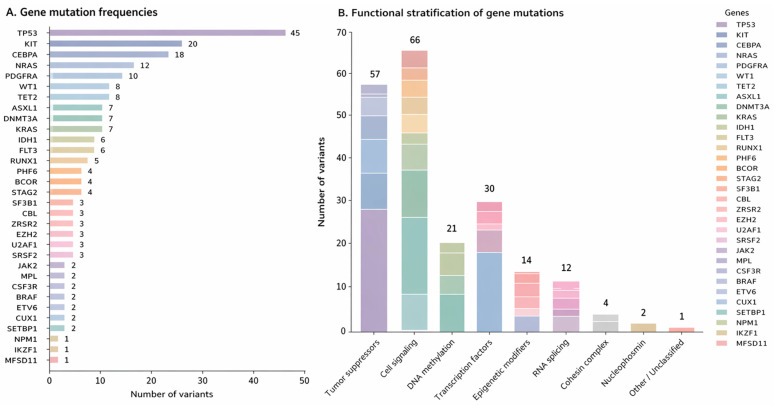
Gene-level mutation frequencies and functional genomic stratification in AML (n = 104). (**A**) Gene mutation frequencies: Horizontal bar plot showing the absolute number of variants detected per gene across the cohort. Genes are ordered in descending frequency, highlighting the most recurrently mutated genes. (**B**) Functional stratification of gene mutations: Stacked bar chart depicting the aggregation of variants according to predefined functional gene groups, with individual gene contributions represented within each bar. The total number of variants within each functional category is indicated above the bars.

Functional classification demonstrated a predominance of cell-signaling pathway alterations (62 variants; 30.4%), largely driven by *FLT3*, *NRAS*, and *KRAS*. Tumor suppressor genes accounted for 55 variants (27.0%), with *TP53* and *WT1* being the most frequently affected. Transcription-factor genes, principally *RUNX1*, *CEBPA*, and *ETV6*, harbored 28 variants (13.7%). RNA-splicing genes (*SRSF2*, *U2AF1*, *SF3B1*, and *ZRSR2*) comprised 15 variants (7.4%), while epigenetic modifiers (*ASXL1*, *BCOR*, *EZH2*, and *BCORL1*) contributed 14 variants (6.9%). In contrast, spliceosome-associated and cohesin complex gene alterations were infrequent. The Oncoprint in Figure 4 visualizes gene-level somatic variants across 104 AML patients, annotated by variant allele frequency (VAF), mutation type, and functional gene groups, while Figure 5 summarizes the frequency and functional stratification of our cohort.

### 2.8. Variant Allele Frequency (VAF) Landscape

**Figure 6:**
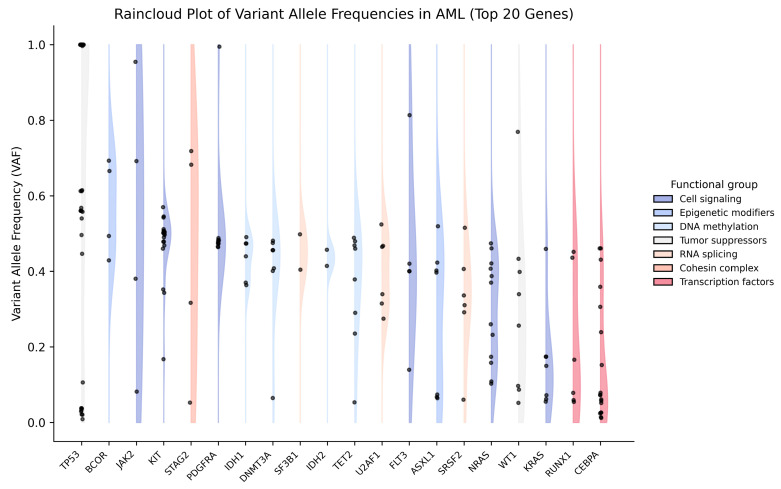
Distribution of variant allele frequencies (VAF) across recurrently mutated genes in AML (top 20 genes). Each vertical panel represents an individual gene, with genes arranged along the x-axis and VAF values plotted on the y-axis (range: 0–1). Genes are color-coded according to their functional classification, including cell-signaling, DNA methylation, epigenetic modifiers, tumor suppressors, RNA splicing, cohesin complex, and transcription factors, as indicated in the legend.

VAF analysis showed marked inter-gene and inter-patient heterogeneity, with an overall mean cohort VAF of 0.42. Tumor suppressor and epigenetic regulator mutations (*TP53*, *DNMT3A*, *IDH1/2*, *TET2*, *ASXL1*) dominated the moderate-to-high VAF range, consistent with early clonal events, with *TP53* displaying a characteristic bimodal pattern as shown in Figure 6. In contrast, *RAS* pathway and transcription-factor mutations (*KRAS*, *NRAS*, *RUNX1*, *CEBPA*) were largely low-VAF, while signaling, cohesin, and RNA-splicing genes showed intermediate and heterogeneous clonal behavior.

### 2.9. Co-Mutation Architecture and Gene-Level Correlation Patterns

**Figure 7:**
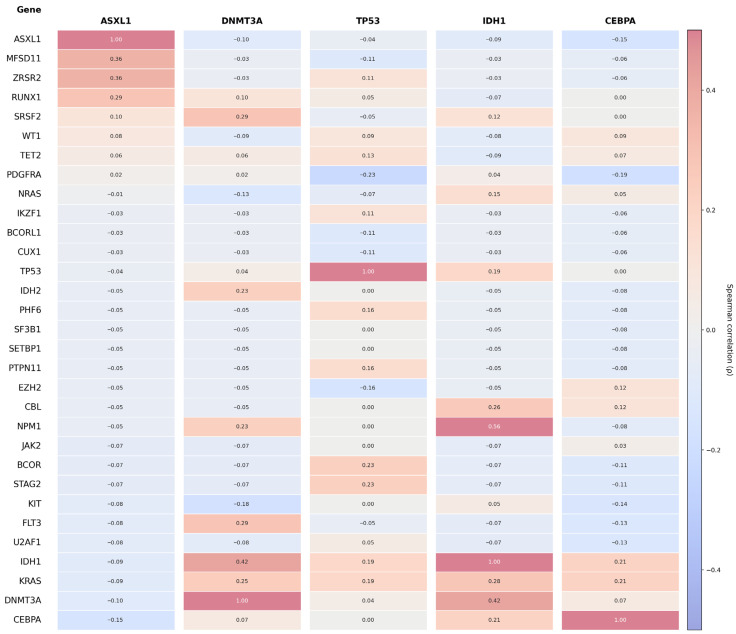
Gene-level co-mutation patterns and correlation structure in AML based on Spearman correlation analysis. Columns represent selected index genes (*ASXL1*, *DNMT3A*, *TP53*, *IDH1*, and *CEBPA*), while rows include a broader set of recurrently mutated genes. Each cell displays the correlation coefficient (ρ), with corresponding color intensity ranging from blue (negative correlation) to red (positive correlation), as indicated by the color scale.

**Figure 8:**
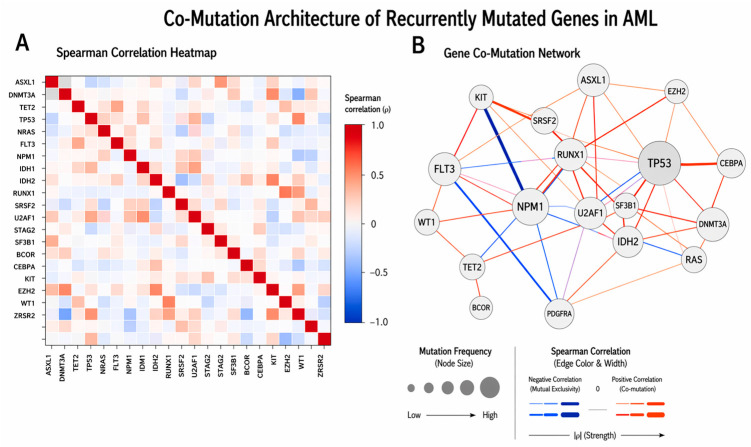
Co-Mutation Architecture of Recurrently Mutated Genes in AML. (**A**) Spearman correlation heatmap: Pairwise relationships between recurrently mutated genes are quantified using Spearman’s rank correlation coefficient (ρ) and visualized as a symmetric heatmap. Each row and column represents a gene, with diagonal values (ρ = 1.0) indicating self-correlation. The color scale ranges from blue (negative correlation) to red (positive correlation), reflecting the strength and direction of association. (**B**) Gene co-mutation network: Network graph representing significant or notable gene–gene associations derived from correlation analysis. Each node represents a gene, with node size proportional to its mutation frequency, highlighting highly recurrently mutated genes. Edges represent pairwise associations, with red/orange edges indicating positive correlations (co-mutation) and blue edges indicating negative correlations (mutual exclusivity). Edge thickness reflects the strength of the association.

The Spearman correlation heatmap demonstrated selective positive associations, most prominently for *DNMT3A–IDH1* (ρ ≈ 0.43) as depicted in Figure 7, *BCOR–STAG2*, *BCOR–U2AF1*, and *JAK2–SRSF2* (ρ ≈ 0.41–0.48), with moderate correlations observed for *KRAS–IDH1* (ρ = 0.28), *KRAS–DNMT3A* (ρ = 0.25), *RUNX1–ASXL1* (ρ = 0.29), and *FLT3–DNMT3A* (ρ = 0.29), whereas *KIT*- and *PDGFRA*-associated pairs demonstrated weak or negative correlations, consistent with relative mutational independence as seen in Figure 8.

### 2.10. Functional Interaction Networks

**Figure 9:**
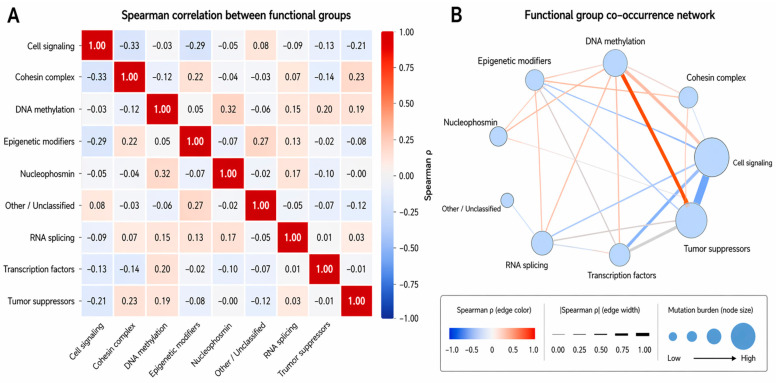
Functional group–level co-mutation architecture in AML. (**A**) Spearman correlation heatmap of functional groups: Heatmap showing pairwise associations between functional categories. Each cell represents the Spearman correlation coefficient (ρ) calculated based on the co-occurrence of mutations within the same patients. Color intensity reflects the strength and direction of association, ranging from blue (negative correlation) to red (positive correlation). (**B**) Functional-group co-occurrence network: Network diagram visualizing interrelationships between functional gene groups. Each node represents a functional category, with node size proportional to the overall mutation burden within that group. Edges represent pairwise associations, where warmer colors (red/orange) indicate positive correlations (co-occurrence) and cooler colors (blue) indicate negative correlations (mutual exclusivity). Edge thickness corresponds to the magnitude of the correlation.

Figure 9 depicts the functional-group analysis revealing non-random mutational architecture in AML. DNA-methylation programs co-occurred with tumor suppressors (OR ≈ 4.6, *p* = 0.007), transcription factors (OR ≈ 3.9, *p* = 0.023), and *NPM1* (ρ = 0.32), with additional positive correlations for transcription factors (ρ = 0.20) and tumor suppressors (ρ = 0.19). Cohesin mutations showed modest coupling with tumor suppressors (ρ = 0.23) and epigenetic modifiers (ρ = 0.22). In contrast, cell-signaling alterations were depleted in epigenetic-modifier–mutant cases (OR ≈ 0.17, *p* = 0.006) and demonstrated inverse associations with cohesin (ρ = −0.33), epigenetic modifiers (ρ = −0.29), and tumor suppressors (ρ = −0.21). Burden-based analysis confirmed an inverse signaling–epigenetic axis (ρ = −0.33; FDR q = 0.072), with network modeling identifying DNA methylation as a central hub and signaling pathways as a largely independent molecular trajectory.

### 2.11. Cytogenetic Spectrum of AML Patients

Cytogenetic analysis was available in 88/104 (85%) patients. A normal karyotype was the most frequent finding, observed in 38 patients (43.2%). Recurrent AML-defining abnormalities were identified in 21 patients (23.9%), comprising t(8;21), inv(16)/t(16;16), and t(15;17) (each 6.8%), and *KMT2A* rearrangements (3.4%). AML-MR–defining cytogenetic abnormalities were present in 14 patients (15.9%), with complex karyotype being the most common (6.8%), followed by −7/del(7q) (2.3%). Other AML-MR–defining abnormalities including −5/del(5q), −13/del(13q), del(11q), del(12p), i(17q), idic(X)(q13) were each observed in 1.1%. Numerical abnormalities were seen in 14 patients (15.9%), most frequently trisomy 8 (10.2%), while other gains/losses (+13, +19, +21, +22, −Y) were rare (1.1% each). Non-defining structural abnormalities were uncommon, with del(9q) identified in a single patient (1.1%).

### 2.12. Pediatric AML Patients

Among the cohort of 104 patients, 9 (8.7%) were of the pediatric age group with ages ranging from 5 to 13 years, and a predominance of males (7/9, 77.8%). Among them, molecular profiling revealed pathogenic or likely pathogenic variants in four patients, while 7 out of these 9 patients (77.8%) also had recurrent cytogenetic abnormalities. The molecular and cytogenetic profile of our pediatric AML patients is outlined in Table 1.

## 3. Discussion

In this study, we recruited 104 newly diagnosed AML patients and performed targeted NGS using a 58-gene panel. Overall, around 77% of our patients harbored at least one somatic variant, while 23% did not show any somatic variant. The frequency of mutations detected in AML patients is variable in the literature, depending on the coverage of different NGS panels used. In a Chinese cohort of 87 AML patients, targeted NGS using a panel covering 120 genes related to myeloid disorders revealed 89.7% of the patients had somatic variants [13]. Another study using a panel of 19 genes showed that variants were detected in approximately 80% of their AML patients [14]. Thus, the diagnostic yield depends on the depth and coverage of genes under investigation.

The mean mutational burden of 1.96 variants per patient, with most cases harboring three or fewer mutations, closely mirrors international AML experience when analysis is limited to recurrent driver genes on targeted or clinical sequencing panels. Large genomic studies have consistently shown that, despite the presence of additional background mutations detectable by whole-genome or whole-exome sequencing, AML is typically driven by a small number of biologically meaningful lesions, most commonly involving epigenetic regulators, tumor suppressors, and signaling pathways [4]. Data from TCGA and subsequent large AML cohorts confirm that most patients carry only a limited set of recurrent driver mutations, with higher mutational burden confined to specific high-risk subgroups [15,16]. A transition-to-transversion ratio (Ti/Tv) of 1.27:1 is indicative of typical somatic mutagenesis, predominantly C>T transitions (33.7%) as previously described in AML and other hematologic malignancies [17].

Variant allele frequencies helped clarify clonal timing. High-VAF mutations in *TP53*, *DNMT3A*, *IDH1/2*, and *TET2* likely represent early founder events, while low-VAF *RAS* and transcription-factor mutations appear to arise later during subclonal evolution. The bimodal VAF pattern of TP53 highlights its role both in disease initiation and subsequent clonal expansion. Although these cases represent de novo AML, delayed diagnosis in an under-resourced setting and referral-center bias likely result in presentation at more advanced stages. Despite this, the clonal hierarchy observed closely parallels international AML models, in which epigenetic and *TP53* alterations arise early and activating signaling lesions are acquired later under selective pressure.

More than half of the detected variants were clinically relevant (54.9%), consistent with contemporary NGS-based AML studies demonstrating a high burden of pathogenic and likely pathogenic driver alterations that increasingly define the clinically actionable subset.

Cell-signaling mutations were the most frequent (38.4%), consistent with established AML models where these alterations drive leukemic proliferation. However, their relative enrichment in this cohort suggests a greater reliance on MAPK pathway activation. Notably, recent 2026 data indicate that NRAS–CEBPA co-mutation is associated with significantly poorer survival (HR ~3.1), underscoring a potentially high-risk cooperative biological interaction.

More than half of the detected variants were clinically relevant (54.9%), consistent with contemporary NGS-based AML studies demonstrating a high burden of pathogenic and likely pathogenic driver alterations that increasingly define the clinically actionable subset [18]. Cell-signaling mutations were the most frequent (38.4%), consistent with established AML models where these alterations drive leukemic proliferation. However, their relative enrichment in this cohort suggests a greater reliance on *MAPK* pathway activation [19]. Notably, recent 2026 data indicate that *NRAS–CEBPA* co-mutation is associated with significantly poorer survival (HR ~3.1), underscoring a potentially high-risk cooperative biological interaction [20]. *CEBPA* mutations were seen in 8.0% of cases, which is in line with what has been reported internationally (~7–20%) and reflects a similar genomic pattern to global AML cohorts; importantly, certain *CEBPA* mutations (particularly bZIP or double mutations) are associated with a more favorable risk profile [21]. In contrast, *WT1* mutations were identified in 7.1% of cases; again, within the expected range (~10%); and are well known to be linked with poorer outcomes, including a higher risk of relapse and overall adverse prognosis [22].

We found a high frequency of benign variants in the *KIT* and *PDGFRA* genes. According to existing literature, many mutations in AML are benign, ‘passenger’ mutations that were pre-existing in the cell as part of normal aging, and do not contribute to the disease process itself [23]. These are detected as synonymous variants during sequencing.

Among the pediatric subgroup of our AML cohort, the most frequent pathogenic variants were of the *NRAS* and *FLT3* genes. A study from the Children’s Oncology Group found that up to 10% of pediatric AML patients harbor *NRAS* mutations [24]. Similarly, *FLT3* mutations are reported in 10 to 15% of childhood AML cases and are associated with poor prognosis [25]. We also found a single case of pediatric AML harboring a pathogenic variant of *PHF6*. *PHF6* variants are a rare occurrence in childhood AML, and their prognosis remains poorly understood [26]. Such cases require longitudinal follow-up for a better understanding of patient outcomes.

This study presents the first comprehensive mapping of somatic mutational patterns in a Pakistani AML cohort using NGS. The findings provide population-specific genomic data to inform diagnostic, prognostic, and therapeutic strategies. The strength of this study lies in its robust sample size and the use of a well-established NGS panel and bioinformatics pipeline. The findings provide population-specific genomic data to inform diagnostic, prognostic, and therapeutic strategies. Limitations include the lack of germline controls and the absence of outcome data, which remain essential to validate prognostic associations. Another limitation is the exclusion of secondary AML cases, which may limit the applicability of findings to the full spectrum of AML biology.

Our cohort reflects a real-world AML population from an under-resourced healthcare setting. Limited access to care leads to late presentation, often with advanced and biologically complex disease. As a national tertiary referral center, AFIP receives many diagnostically challenging AML cases, enriching the cohort for high-risk disease. This referral bias likely explains the higher mutational complexity and increased burden of adverse-risk genomic alterations observed, highlighting the distinct AML landscape in low- and middle-income countries.

## 4. Materials and Methods

### 4.1. Study Design and Setting

This prospective, observational cross-sectional study was conducted from July 2021 to July 2025 at the Department of Molecular Hematology, Armed Forces Institute of Pathology (AFIP), Rawalpindi, Pakistan.

### 4.2. Patient Selection and Eligibility Criteria

Newly diagnosed patients of any age or gender with de novo AML were enrolled. Patients with prior chemotherapy or radiotherapy, or secondary AML, were excluded. Diagnosis was established according to WHO 2022 criteria using integrated morphology, immunophenotyping, cytogenetics, and molecular profiling. Written informed consent was obtained, and clinical, laboratory, cytogenetics, and NGS data were prospectively recorded on a standardized proforma.

### 4.3. Cytogenetics and FISH Analysis

Conventional cytogenetic analysis was performed on bone marrow samples (3 mL, sodium heparin) using standard G-banding (Giemsa–trypsin) techniques. Karyotypes were interpreted in accordance with the International System for Human Cytogenetic Nomenclature (ISCN), with a minimum of 20 metaphases analyzed per case using the CytoVision (v4.5.2-v4.5.4 and v7.3.1-7.3.2) semi-automated imaging platform.

Interphase FISH was conducted on peripheral blood and/or bone marrow specimens following standard processing protocols. Locus-specific probes targeting KMT2A, MECOM, and NUP98 (MetaSystems v3.12.9) were applied, and ≥500 interphase nuclei per probe set were evaluated using fluorescence microscopy with appropriate dual-color filters.

### 4.4. Sample Collection, DNA Extraction, and Quality Assessment

Peripheral blood and/or bone marrow samples were collected in EDTA at diagnosis, with adequate blast representation in all analyzed specimens. Samples were not subjected to mononuclear cell separation; genomic DNA was extracted directly from the samples using the Thermo Fisher GeneJET Genomic DNA Purification Kit (ThermoFisher Scientific, Baltics UAB, Lithuania). DNA integrity was assessed by agarose gel electrophoresis, with storage at 4 °C (short term) and −20 °C (long term). DNA quantity was measured using the Qubit dsDNA High Sensitivity assay, targeting 50–100 ng for library preparation. DNA purity was assessed on an Inno-M microplate spectrophotometer (LTek Co., Ltd., Seongnam, South Korea), with acceptable A260/A280 ratios of 1.6–2.0.

### 4.5. Targeted NGS Panel and Library Preparation

DNA libraries were prepared using highly multiplexed oligonucleotide probes targeting 58 genes, comprising 18 full genes and exonic hotspots of 40 genes. The panel covers 107.9 kb of genomic content with 766 amplicons. Following the extension-ligation reaction, the DNA templates were PCR amplified. Unique sample-specific indexes were added to each library during PCR to facilitate the pooling of libraries. The final PCR products were processed into a single-stranded, adapter-ligated, normalized library using a bead-based protocol.

### 4.6. Next-Generation Sequencing Workflow

Sequencing was performed on an Illumina MiSeq platform (Illumina, San Diego, CA, USA), utilizing sequencing-by-synthesis (SBS) chemistry for accurate and high-throughput base-calling. Libraries were loaded into reagent cartridges, and sequencing runs included quality control samples to verify the accuracy and reproducibility of the results. Raw sequencing data were processed using MiSeq Reporter software (v2.6.2.3). The data were demultiplexed, and FASTQ files were generated for individual samples.

### 4.7. Bioinformatics and Variant Interpretation

FASTQ files were uploaded on Illumina Connected Analytics (ICA) software (v2.0), and the Illumina DRAGEN (Dynamic Read Analysis for GENomics) bioinformatics platform (v4.3.6; Illumina, San Diego, CA, USA) was used for further processing. Reads were aligned to the human reference genome (GRCh37/hg19) to produce BAM files. Variant calling format (VCF) files were generated, which were subsequently analyzed on the Illumina Connected Insights (ICI) (version 5.2.3; Illumina, San Diego, CA, USA). ICI performs variant annotation and filtration incorporating information from various databases such as 1000 Genomes project, gnomAD, dbSNP, ClinVar, COSMIC, OncoKB, etc. Variants were finally reported according to standardized guidelines given by the consensus recommendations of the Association of Molecular Pathology (AMP), College of American Pathologists (CAP), and American Society of Clinical Oncology (ASCO) [12].

### 4.8. Statistical Analysis

Data analysis was performed using Microsoft Excel and the Statistical Package for Social Sciences (SPSS) version 23.

### 4.9. Ethics Approval and Consent

Ethical approval was obtained from the Institutional Review Board AFIP Rawalpindi in accordance with the Declaration of Helsinki prior to study initiation. Written informed consent for participation in the study was obtained from all patients prior to enrollment.

## 5. Conclusions

In summary, this study provides the first high-resolution map of AML genomics in Pakistan, demonstrating a landscape driven by signaling genes (NRAS, KRAS), with additional contributions from transcriptional (CEBPA, RUNX1) and epigenetic regulators (TET2, ASXL1, IDH1), reflecting a convergent multi-pathway genomic architecture. Distinct clonal hierarchies and cooperative mutational networks differentiate this profile from Western datasets, underscoring the importance of region-specific genomic data for accurate risk stratification and therapeutic guidance. Collectively, these findings highlight AML heterogeneity and support integrated genomic profiling for precision-directed management.

## Figures and Tables

**Table 1:** Cytogenetic and molecular profile of pediatric AML patients. This table presents the cytogenetic abnormalities and next-generation sequencing (NGS) findings in pediatric AML patients (n = 9). Cytogenetic data include recurrent AML-defining rearrangements, while NGS results list detected variants classified as pathogenic, likely pathogenic, or benign. Cases with no detectable variants are indicated accordingly.

Patient No.	Age (Years)	Gender	Cytogenetics	Variants on NGS
1	5	M	t(9;11)(p22;q23)	No variant reported
2	6	F	Not available	*KIT* (benign)
3	8	M	t(10;11)(p12;q23)	*KIT* (benign), *FLT3* (likely pathogenic), *PHF6* (pathogenic)
4	8	M	t(8;21)(q22;q22.1)	No gene variant reported
5	9	M	t(8;21)(q22;q22.1)	No gene variant reported
6	9	M	t(5;11)(q35;p15.5)	*KIT* (benign), *FLT3* (likely pathogenic)
7	11	M	t(16;16)(p13.1;q22.1), del(7q)	*NRAS* (pathogenic), *SETBP1* (likely pathogenic)
8	11	F	Not available	*TP53* (2 pathogenic variants), *KIT* (benign), *NRAS* (pathogenic), *IKZF1* (likely pathogenic)
9	13	M	t(9;11)(p22;q23)	No gene variant reported

## Data Availability

The data that support the findings of this study are available from the corresponding author upon reasonable request.
